# Public reporting improves antibiotic prescribing for upper respiratory tract infections in primary care: a matched-pair cluster-randomized trial in China

**DOI:** 10.1186/1478-4505-12-61

**Published:** 2014-10-10

**Authors:** Lianping Yang, Chaojie Liu, Lijun Wang, Xi Yin, Xinping Zhang

**Affiliations:** School of Medicine and Health Management, Tongji Medical College of Huazhong University of Science and Technology, No.13. Hangkong Road, Wuhan, Hubei province 430030 PR China; School of Public Health, La Trobe University, 1 Kingsbury Dr, Bundoora, Victoria, 3086 Australia

**Keywords:** Antibiotics, China, Primary care, Public reporting, Upper respiratory tract infection

## Abstract

**Background:**

Inappropriate use and overuse of antibiotics is a serious concern in the treatment of upper respiratory tract infections (URTIs), especially in developing countries. In recent decades, information disclosure and public reporting (PR) has become an instrument for encouraging good practice in healthcare. This study evaluated the impact of PR on antibiotic prescribing for URTIs in a sample of primary care institutions in China.

**Methods:**

A matched-pair cluster-randomized trial was undertaken in QJ city, with 20 primary care institutions participating in the trial. Participating institutions were matched into pairs before being randomly assigned into a control and an intervention group. Prescription statistics were disclosed to patients, health authorities, and health workers monthly within the intervention group, starting from October 2013. Outpatient prescriptions for URTIs were collected from both groups before (1^st^ March to 31^st^ May, 2013) and after the intervention (1^st^ March to 31^st^ May, 2014). A total of 34,815 URTI prescriptions were included in a difference-in-difference analysis using multivariate linear or logistic regression models, controlling for patient attributes as well as institutional characteristics.

**Results:**

Overall, 90% URTI prescriptions required antibiotics and 21% required combined use of antibiotics. More than 77% of URTI prescriptions required intravenous (IV) injection or infusion of drugs. PR resulted in a 9 percentage point (95% CI -17 to -1) reduction in the use of oral antibiotics (adjusted RR =39%, *P* =0.027), while the use of injectable antibiotics remained unchanged. PR led to a 7 percentage point reduction (95% CI -14 to 0; adjusted RR =36%) in combined use of antibiotics (*P* =0.049), which was largely driven by a significant reduction in male patients (-7.5%, 95% CI -14 to -1, *P* =0.03). The intervention had little impact on the use of IV injections or infusions, or the total prescription expenditure.

**Conclusions:**

The results suggest that PR could improve prescribing practices in terms of reducing oral antibiotics and combined use of antibiotics; however, the impacts were limited. We suggest that PR would probably be enhanced by provider payment reform, management and training for providers, and health education for patients.

## Background

Inappropriate use and overuse of antibiotics is a serious concern, especially in developing countries such as China [1]. It is perhaps the most prominent manifestation of irrational drug prescribing and contributes to bacterial resistance, which is an emergent threat to the global population [2–4]. Nosocomial or hospital-acquired infection of antibiotic-resistant bacteria has the capacity to increase medical costs, length of hospital stay, and ultimately patient mortality [5, 6].

Upper respiratory tract infections (URTIs) are one of the most common health conditions seen by primary care workers, and the associated overuse of antibiotics is widespread [7]. In the UK, for example, general practitioners prescribe antibiotics to 67% of patients presenting with respiratory infections, including 47% of those with URTIs [8]. It was estimated that 60% of respiratory infections prescribed with antibiotics in the UK are self-limiting conditions [9]. A large cohort study of antibiotic usage associated with URTI in UK primary care settings concluded that prophylactic antibiotic prescriptions are unjustified in reducing the risk of serious complications in URTIs [10].

In China, approximately 75% of patients with seasonal influenza were estimated to be prescribed with antibiotics, more than doubled of the maximum rate (30%) recommended by the World Health Organization (WHO) [1]. Several systematic reviews have shown that antibiotics are of limited effectiveness in the treatment of URTIs [11] and suggested no benefit in colds [12].

Growing public health concerns regarding the implications of inappropriate use and overuse of antibiotics have resulted in global action [13]; China is no exception. The recent health reform (2009–2011) in China articulated specific governance and incentive structures for public health organisations to address the issues of poor quality in prescribing, such as the enforcement of an essential medicine list policy and decoupling the nexus between medicine sales to health worker remuneration and bonus payments in primary care organisations [14]. Despite this, empirical evidence demonstrates that the effectiveness of these policy interventions has been short of expectations [15].

In recent decades, public reporting (PR) has started to attract attention as a vehicle for improving the performance of health organisations [16–20]. The United States has led the PR movement, along with the UK [21]. The rationale behind this approach draws experience from other industries with theoretical support from the domains of psychology, economics, and organizational behaviour. Public release of performance data is considered a bottom-up intervention compared with government regulation [22], and is intended to improve quality of care through imposing a higher level of transparency and accountability [23]. Frølich et al. believe that PR can provide both financial and reputational incentives for health providers to improve quality of care, although contextual factors (at the levels of the markets and provider organisation) and characteristics of providers and patients may enhance or mitigate their responses to those incentives [24]. Reputational incentives could also have financial implications.

This study aims to explore the potential use of PR for improving the quality of prescribing in primary care organisations using a social experiment. The study has important implications, primarily to reduce unnecessary antibiotic prescription, but also as an analysis of whether greater transparency leads to improved healthcare outcomes. Provider-specific comparative performance reporting is currently being debated and implemented in a subset of developed countries, but only in a small group of developing countries [25]. Scant information is available regarding the effectiveness of PR in primary care settings, particularly in relation to prescribing behaviours. The concept of PR is almost completely novel for health workers in China.

## Methods

### Setting

This study was undertaken in QJ city of Hubei province, involving 20 primary care organisations. Hubei has a population of 58 million and a GDP of 2,225 billion (Yuan in 2012), ranking in the middle range of all Chinese provinces. QJ is located in central Hubei, with a land area of 2,004 km^2^ and a population of 1 million. The annual GDP of QJ reached 49.3 billion (Yuan) in 2012, just above the average level of all cities in Hubei. The majority of inhabitants in QJ are of Han ethnicity, with about 8,000 from Hui, Tujia, and other ethnic groups.

We selected QJ due to ready access to prescription data and implementation of intervention measures. Primary care organisations in QJ have the benefit of an electronic health information system (HIS). The QJ government has been very supportive of this project.

Written informed consent was obtained from all participants. This study was approved by the Ethical Review Committee of Tongji Medical College, Huazhong University of Science and Technology (No. IORG0003571).

### Experimental design

This research adopted a matched-pair randomized trial design. The 20 participating primary care organisations were paired according to their size, geographic location and economic status, and population serviced. We considered all the organisational characteristics presented in Table 1 in the pair-matching process. The Technique for Order Preference by Similarity to Ideal Solution (TOPSIS) method was adopted to calculate a summed score from the matching variables (standardised) for each participating organisation. TOPSIS is a multi-criteria decision analysis method that estimates the geometric distance between each alternative and the ideal alternative to a given solution [26]. The participating organisations were ranked in order according to their distances to the positive and the negative ideal alternatives. Adjacent organisations were paired and assigned randomly to the intervention and control groups.

**Table 1:** **Characteristics of participating institutions and patients**

Characteristics	Control group		Intervention group	
**Primary care institutions**				
Sample size	10		10	
Average population serviced (10,000)	4.04 (1.80)		3.83 (1.43)	
Number of beds	65.60 (19.61)		60.00 (21.73)	
Number of doctors	28.30 (7.42)		26.30 (8.54)	
Outpatient visits per year	50199.60 (29236.49)		49108.20 (23171.97)	
Inpatient admission per year	1348.60 (499.95)		1482.20 (703.11)	
Drug sales revenue per year (10,000 Yuan)	188.87 (100.01)		150.78 (49.66)	
Variety of drugs in stock	307.60 (145.97)		377.10 (172.55)	
Percentage (%) of drug sales in total revenue	35.73 (21.52)		25.42 (14.89)	
Average revenue per doctor (10,000 Yuan)	22.46 (8.90)		27.01 (8.94)	
**Patients with prescriptions for URTI**	**Pre-intervention**	**Post-intervention**	**Pre-intervention**	**Post-intervention**
Sample size	7294	10369	4378	12774
Average age (years)	23.41 (22.52)	23.25 (23.73)	22.36 (22.83)	24.39 (24.48)
Proportion of male patients (%)	47.92	51.10	50.18	48.76
Proportion of patients enrolled with NCMS	81.81	87.36	84.65	87.40

Notes: Data are presented as % or mean (standard deviation) unless otherwise stated. The characteristics of primary care organisations were drawn from the 2012 data before the intervention started. NCMS, New Cooperative Medical Scheme; URTI, Upper respiratory tract infection.

Participants in the intervention group and the control group were exposed to the same political, financial, and administrative environments: the only exception being the intervention measures. Identical training on appropriate prescription practice was offered to both intervention and control groups.

### Intervention measures

The intervention measures were designed after considering the following assumptions [21, 27]:

- Patient choice of healthcare providers: patients or potential consumers would make an informed choice of their preferred provider(s) based on reported prescription indicators, exerting consumer pressure on healthcare providers.
- Use of reported data by regulators and managers: medical insurance authorities, local health authorities, and health organisation managers may use the reported data for decisions in payment (or reimbursement), health planning, and performance management, exerting financial and administrative pressure on healthcare providers (individual or institutional level).
- Provider efforts to improve quality of care: physicians and healthcare organisations have intrinsic motivation to improve quality of care, especially if their quality of care falls below average.

The PR intervention started in October 2013. Three elements were included in the PR of this study: prescribing indicators were calculated using data extracted from the HIS on a monthly basis; healthcare providers (at both individual and institution levels) were ranked using the prescribing indicators and the reports were displayed in a public space in the primary care organisations in the intervention group; the monthly performance reports and league tables were submitted to local health authorities and organisational managers in the intervention group (see *Public reporting package* below). The reports also contained a brief explanation about the purpose of the PR intervention (i.e., to curb overuse of antibiotics). Administrative measures (e.g., meetings and newsletters) were taken to ensure that all prescribers in the intervention group were aware of the reports. However, there was no action taken to individually alert patients to the reports, although these reports (including poster displays and brochures) were readily available every day in a designated public space.

#### Public reporting package

1. **Timeline:** Monthly reporting in the intervention group started in October 2013, which is still continuing at the time of writing.
2. **Reported indicators:** Prescription indicators were calculated at both individual (physician) and institutional levels, including:
  Percentage of prescriptions requiring antibiotics (%) = Number of prescriptions requiring antibiotics/total number of prescriptions by a physician (or institution) in one month × 100%.
  Percentage of prescriptions requiring IV injections (%) = Number of prescriptions requiring IV injections/total number of prescriptions by a physician (or institution) in one month × 100%.
  Expenditure per prescription (Yuan) = Total expenditure of prescriptions/total number of prescriptions by a physician (or institution) in one month.

3) **Dissemination:** Prescribing physicians and hospitals were ranked in each of the above indicators. The league tables were publicly available to consumers and health workers alike, displayed on a bulletin board in the lobby of outpatient departments. Hard copies of the reports were also submitted to local health authorities and presidents of the hospitals in the intervention group.
4) **Update of reports:** The reports were updated monthly and made available on the first week of each month.

To ensure compliance with the intervention, we sent trained investigators to the intervention sites at random to monitor the implementation of intervention measures on a monthly basis.

### Data collection

Data pertaining to the characteristics of the participating organisations were extracted through their respective administrative systems prior to commencement of the PR intervention, covering the entire year of 2012. These included population serviced, number of outpatient visits, number of hospital admissions, bed numbers, number of doctors, gross revenue, revenue from drug sales, and revenue from government subsidies. The research team validated these data in interviews with managers and reconciled discrepancies as necessary.

Prescription data were drawn from the electronic HIS. For the purpose of this study, we collected unidentified prescription data from the participating organisations for a period of four months prior to the intervention (1^st^ March to 31^st^ May, 2013) and four months after start of the intervention (1^st^ March to 31^st^ May, 2014). All prescriptions associated with URTI were identified and collated for data analyses. The prescription data also contained demographic information (gender, age, insurance status) of the patients, tests and examinations ordered by the physicians, and expenditure in relation to the prescriptions.

### Statistical analysis

The prescribing indicators used in this study were adapted from the WHO/International Network for the Rational Use of Drugs indicators, which have been used widely [2, 15, 28]. We examined the extent to which the PR intervention changed the prescribing patterns in relation to those indicators, such as:

1. Percentage of prescriptions requiring antibiotics;
2. Percentage of prescriptions requiring two or more antibiotics;
3. Percentage of prescriptions requiring intravenous (IV) injection (peripheral IV administration using a syringe driver [29]);
4. Percentage of prescriptions requiring infusion (medicines are introduced into a vein or between tissues by using an infusion pump or bag [29]);
5. Expenditure per patient visit (including consultations, diagnostic tests, and prescriptions).

A difference-in-difference (DID) estimation of the above indicators was used in our study to assess the impact of the PR intervention on prescribing patterns. The DID method allows us to control confounding influences of independent variables, as well as imbalance between groups in baseline (dependent) variables (due to chances of imperfect randomisation).

We performed regression modelling for estimation of the effect size of PR. We reported both unadjusted estimates of effect size and those adjusted for characteristics of patients (age, sex and enrolment with the New Cooperative Medical Scheme (NCMS)) and hospitals (number of beds, number of doctors, population serviced, outpatient visits a year, inpatient admissions per annum, drug sales revenue per annum, variety of drugs in stock, percentage of drug sales in total revenue, and average revenue per doctor).

For binary dependent variables (in relation to indicator 1, 2, 3, and 4), we used logistic regression models. For a given patient *i* serviced within organisation *j*, the probability p_*ij*_ of the occurrence of a dichotomous outcome y_*ij*_ (i.e., value 1 for a patient receiving antibiotics, 0 otherwise) modelled as a logistic model can be expressed as:
Model1

and report marginal effects, where

- i =1 . . . i patient, j =1. . . j primary care organisation;
- *T*_*ij*_ indicates i^th^ patient receiving a prescription within j^th^ primary care organisation before or after intervention (0 before or 1 after);
- *G*_*ij*_ indicates i^th^ patient receiving a prescription within j^th^ primary care organisation that had or had not implemented PR intervention (0 control or 1 intervention);
- *EFFECT*_*ij*_ = *T*_*ij*_ * *G*_*ij*_ , which measures invention effect and coefficient *β*_*e*_ indicates the effect size;
- o =1. . . o patient visit covariate, p =1. . .p primary care organisation covariate.

For the continuous dependent variable (in relation to indicator 5, log transformed), we used a least squares regression model, which can be expressed as:
Model2

*X*_*oij*_ and *X*_*pij*_ are the control (independent) variables. In addition, a dummy variable was generated to measure the cluster-pair fixed effect. Robust standard errors were clustered at the organisational level.

Coefficient *β*_*e*_ in the regression model 1 and 2 is actually an estimation of the interaction between intervention and time, measuring the DID. For all of the outcome indicators, we conducted subgroup analyses by gender.

All statistical analyses were performed using Stata (version 10).

## Results

### Characteristics of participating organisations and patients

Similar characteristics of participating organisations were shown between intervention and control groups (Table 1). Each organisation served on average a population of 40,000 and provided 50,000 outpatient consultations per annum. A total of 34,815 prescriptions for URTI were deemed eligible for inclusion in the study. Those patients prescribed with antibiotics had a mean age of 23.6 years (Standard deviation =23.5); about half (49.5%) of them were male and over 85.9% were enrolled with the NCMS.

### Prescriptions requiring antibiotics

High rates of antibiotic prescription were noted in both intervention and control groups (Table 2). Prior to the intervention, more than 90% of prescriptions for URTIs contained antibiotics.

**Table 2:** **Percentage (%) of prescriptions requiring antibiotics for upper respiratory tract infections**

Indicators	Control group		Intervention group		Treatment effect ^a^			
	Pre	Post	Pre	Post	Unadjusted (95% CI)	***P***	Adjusted (95% CI)	***P***
**Percentage of prescriptions requiring antibiotics (%)**								
All antibiotics	95.28	94.74	90.57	87.55	-1.48 (-6.42, 3.45)	0.556	-1.93 (-6.61, 2.75)	0.419
Oral	25.00	24.63	50.98	40.20	-8.93 (-28.71, 10.86)	0.377	**-9.21 (-17.36, -1.07)**	**0.027**
Injection	80.25	80.00	48.54	55.57	6.02 (-10.81, 22.86)	0.483	4.04 (-5.53, 13.62)	0.407
For male patients	95.57	95.00	90.76	87.56	-1.52 (-6.55, 3.51)	0.553	-2.09 (-6.70, 2.52)	0.374
For female patients	95.03	94.48	90.37	87.53	-1.38 (-6.39, 3.63)	0.589	-2.29 (-6.59, 2.01)	0.296
**Percentage of prescriptions requiring two or more antibiotics (%)**								
All	16.56	22.21	21.68	23.61	-4.25 (-13.62, 5.12)	0.374	**-6.97 (-13.94, 0.00)**	**0.049**
For male patients	16.08	22.17	22.21	22.72	-5.11 (-14.09, 3.87)	0.265	**-7.51 (-14.27, -0.74)**	**0.030**
For female patients	17.00	22.22	22.5	24.43	-3.49 (-13.69, 6.71)	0.502	-6.46 (-14.07, 1.15)	0.096

Note: data in bold indicate *P* <0.05; 95% CI, 95% confidence interval.

^a^Percentage point changes derived from DID regression models.

The PR intervention led to only a slight reduction in the use of antibiotics, in particular for oral administration of antibiotics: a 9 percentage point reduction in oral administration of antibiotics was shown (*P* <0.05).

Paradoxically, there was an increase in the percentage of prescriptions requiring two or more antibiotics in both intervention and control groups; however, this increase was 7 percentage points less in the intervention group compared with that in the control group (*P* <0.05). This mainly occurred for male URTI patients (*P* =0.03), whereas the effect was statistically insignificant for female patients.

### Prescriptions requiring IV injection or infusion

No significant differences were found between the intervention and control groups in the changes in percentage of prescriptions requiring IV injection or infusion (Table 3). Overall, the percentage of prescriptions requiring IV injection or infusion remained high during the period of study, with 77% to 90% of prescriptions for URTI requiring IV injection or infusion.

**Table 3:** **Percentage (%) of prescriptions requiring IV injections and infusions and average expenditure per prescription for upper respiratory tract infection**

Indicators	Control group		Intervention group		Treatment effect ^a^			
	Pre	Post	Pre	Post	Unadjusted (95% CI)	***P***	Adjusted (95% CI)	***P***
**Percentage of prescriptions requiring IV injection (%)**								
All	90.00	86.17	81.82	77.89	1.66 (-5.50, 8.83)	0.649	1.23 (-3.82, 6.28)	0.633
Male	90.01	85.75	82.29	78.30	2.05 (-5.77, 9.86)	0.608	1.36 (-4.68, 7.39)	0.659
Female	89.97	86.62	81.34	77.49	1.22 (-5.62, 8.08)	0.725	1.22 (-3.18, 5.62)	0.587
**Percentage of prescriptions requiring infusion (%)**								
All	89.76	85.87	81.45	77.64	1.82 (-5.59, 9.23)	0.630	1.37 (-3.93, 6.67)	0.612
Male	89.81	85.39	81.83	78.02	2.38 (-5.52, 10.27)	0.555	1.72 (-4.32, 7.76)	0.577
Female	89.71	86.39	81.06	77.28	1.20 (-6.00, 8.41)	0.743	1.14 (-3.73, 6.02)	0.645
**Average expenditure per prescription (¥ Yuan)**								
All	27.53	41.21	29.72	37.82	-12.80 (-41.77, 16.17)	0.363	-1.21 (-16.33, 13.90)	0.867
Male	27.60	40.38	29.39	37.74	-10.10 (-38.04, 17.85)	0.455	0.60 (-14.37, 15.57)	0.933
Female	27.46	42.09	30.06	37.89	-15.64 (-45.97, 14.69)	0.290	-3.00 (-18.76, 12.77)	0.692

^a^Percentage point changes or percentage changes derived from DID regression models.

### Average expenditure per visit

No significant differences were found between the intervention and control groups in average expenditure per visit (Table 3). Overall, the average expenditure per visit for URTI patients was low, ranging from 27 to 41 Yuan (roughly USD$5–7). The average expenditure per visit measured in the post-intervention period increased slightly compared to that in the pre-intervention period in both intervention and control groups. Such increase was 10% to 15% less in the intervention group than in the control group. However, the adjusted estimate of treatment effect was close to zero after controlling for independent variables.

## Discussion

PR can have an impact on prescription patterns in primary care settings. This study demonstrated that PR interventions reduced the overall number of prescriptions associated with oral administrated antibiotics and retarded an anticipated potential rise in combined use of two or more antibiotics for URTIs. Although clinical outcomes of such a change were not explored in this study, it is reasonable to expect a positive outcome. Overuse of antibiotics is a widespread and serious problem in China [15, 28, 30], despite clinical guidelines for URTIs that advise against routine use of antibiotics [31]. Systematic reviews with meta-analyses of randomised controlled trials have shown minimal benefit associated with antibiotic prescriptions for URTIs [12, 32, 33]. Meanwhile, there are growing concerns about the possibility of antimicrobial resistance induced by irrational use of antibiotics. It was estimated that abuse of antibiotics for URTIs in low- and middle-income countries contributes to an additional 36% of medical costs [34]. Reducing irrational use of antibiotics can preserve these resources for more cost-effective interventions.

It is important to note that the overall use of antibiotics for URTIs in the participating primary care organisations remained at a high level despite the PR interventions. In addition, the PR interventions failed to show a significant impact on prescriptions using parenteral administration (injection and infusion). This study found that over 87% of prescriptions for URTIs contained antibiotics, much higher than the WHO recommendations [1]. The percentage of prescriptions requiring administration via IV injection and infusion for URTI reached a staggering level – over 77%. Arguably, IV injection and infusion are more likely to impose health risks for the public, leading to iatrogenic and nosocomial problems such as transmission of blood-borne viruses and infection of infusion sites [3].

The persistent high prevalence of antibiotic misuse probably reflects both inertia in physician practice and continued demand from patients. Nevertheless, many Chinese patients regard parenteral administration as integral to high-quality care [3]. The national zero-mark-up policy, introduced during 2009–2011, prohibits primary care organisations from generating excessive profits from retailing drugs, with the intention of making drugs more financially accessible. Health providers are wily and resourceful: fees can be charged in relation to injection or infusion services [35]. This may explain why the apparent consumer demand for antibiotics continues to be high, and why prescribing changes were mainly limited to oral antibiotics.

PR interventions did not have a significant effect on the average expenditure per visit. One plausible explanation could be that the expenditure had already been low due to a range of policies. The participating primary care organisations in both intervention and control groups had been exposed to the same policy environments over the past decade. Primary care organisations are only allowed to stock and provide drugs listed in the essential medicines list; these drugs can only be purchased through a regional centralized tendering and purchasing system at an agreed price; patients (and insurers) are charged with exactly the same purchasing price for their prescribed drugs. Empirical evidence showed that those policies have led to a significant reduction in drug expenditure [15]. Understandably, the capacity for further reduction of drug expenditure in primary care organisations is therefore limited.

There are policy implications arising from this study. Evidence from randomised control trials is regarded as the most robust and reliable. It is rare to find a study that uses a randomised control trial design to test the effect of PR interventions on physician prescribing practices within a complex health system and policy environment [16, 17, 21].

Internationally, healthcare systems are moving towards greater transparency and accountability [22]. Performance reporting in healthcare has been shown to improve the health systems of developed countries [16, 17, 21]. Despite this, reporting systems must be properly developed, considering a system-wide stakeholder participation, a change of organisational culture, and the accuracy and relevancy of reported data. It is also important to be mindful of minimising unintended consequences [36].

PR of prescription performance may impose “pressures” on prescribers arising from consumers, payers, managers, and colleagues. The magnitude of the pressure depends on the level of PR awareness and how the population understands it [24, 27, 37, 38].

We fear that consumer pressure to reduce irrational antibiotic and injectable prescription is limited in China, as the disclosed prescription indicators are difficult for consumers to understand due to limited health literacy. It is unlikely that the general public are either able or inclined to use the reported information to identify and choose healthcare providers. Previous studies show that consumer health literacy is low in China [39]; overuse of antibiotics is common; and physicians may be under pressure to prescribe more, not fewer, antibiotics [1]. The effect of PR on patients may also be jeopardised by limited exposure of patients to PR.

Pressure from payers is usually linked with financial incentives (or disincentives) [24, 40]. In this study, we found that the PR interventions are more likely to have an impact on the use of oral administered antibiotics compared with those administered via IV injection or infusion. This is perhaps because there are no financial incentives directly linked to reduction in irrational use of antibiotics, and fewer prescriptions of oral antibiotics (with zero-profit attached) are less likely to have a negative consequence on organisational revenues than reduced prescriptions of injected antibiotics (where a service fee can be charged). Clearly, these are demonstrable examples of perverse incentives.

Pressures from managers and colleagues may be a major driving force for the impact of PR interventions on prescribers. The working environment of physicians in China is characterised by strong administrative interventions from the government and organisations [41, 42]. Physician buy-in of PR is also essential for success; after all, they make the final prescription decision. Our experience, together with studies conducted elsewhere [43], show that physicians may challenge the validity of the PR and express concerns about performance reporting at individual levels for potential misinterpretations from patients. If a physician believes that the reported data are flawed (e.g., lack of adequate risk adjustment), he or she may be less likely to be influenced by the data. To make PR more acceptable, simplified data presentation, continued risk-adjustment refinement, and internal review before PR are necessary [43].

The development of an adequate PR system should be tailored to the macro- and micro-environment in which the healthcare organisations are operating [40]. Consumers and health care providers should be involved in the design and specification of information requirements, reporting approach, and communication strategies [23]. An ideal PR system should be able to assist patients or potential users to make better informed decisions [44]. Such a system should also address concerns from physicians in order to gain their acceptance [43]. A high quality information system is also critical for easy access and quality assurance of data. Translating good practice from one program to others is a practical approach for building high quality information systems [45]. Nevertheless, none of these measures are likely to take place if the income for services remains as a fee for service, and opportunities remain that maximise income through unnecessary drug administration practices.

### Limitations

This study was undertaken during an early stage of PR interventions, which may underestimate the true impact of the interventions. PR does not have a direct impact on prescriber behaviours. It may take time for healthcare providers to understand the rationale underlying the PR interventions and subsequently to change management and clinical practices. The mechanisms and long term effect of PR on prescribing behaviours warrant further studies.

The positive but limited effect of PR on prescription outcomes might also be indicative of shortfalls in project design and implementations. For example, this project included a mixed reporting system at both organisational and individual levels, which prevents us from exploring further mechanisms of the effect. Nevertheless, the study has provided important evidence for further improving the reporting system.

## Conclusions

This study demonstrated that PR interventions reduced the incidence of oral antibiotic prescription and slowed down the increase of combined use of antibiotics for URTIs. However, this effect is limited. Overall, the use of antibiotics remained at high levels, and the PR interventions failed to make an influence on the frequent use of injections and infusions as an administration route. The impact of PR interventions is dependent upon pressures from consumers, payers, managers, and colleagues. Patient empowerment mechanisms, financial incentives, reliable information systems, consumer education, and prescriber buy-in are required to maximise the impact of PR interventions.

## Abbreviations

DID: Difference-in-difference
HIS: Health information system
IV: Intravenous injection
NCMS: New Cooperative Medical Scheme
PR: Public reporting
TOPSIS: Technique for Order Preference by Similarity to Ideal Solution
URTIs: Upper respiratory tract infections
WHO: World Health Organization.
